# Synthesis and Anticonvulsant Activity Evaluation of 3-alkoxy-4-(4-(hexyloxy/heptyloxy)phenyl)-4*H*-1,2,4 -triazole

**Published:** 2015

**Authors:** Ying-Quan Fang, Chun-Ling Sun, Da-Chuan Liu, Shi-Ben Wang, Zhe-San Quan

**Affiliations:** a *College of Pharmacy, Yanbian University, No. 977, Park Road, Yanji, Jilin, 133002, China.*; b *Department of Pharmacy，ChongqingThree Gorges Medical College, No.366, Tianxing Road, Baian Ba, Wanzhou, Chonqing, 404120, China.*

**Keywords:** Synthesis, Triazole, Triazolone, Anticonvulsant activity, Neurotoxicity, Pentylenetetrazole, 3-Mercaptopropionic acid, Bicuculline

## Abstract

A series of 3-alkoxy-4-(4-(hexyloxy/heptyloxy) phenyl)-4*H*-1,2,4-triazole was synthesized. The anticonvulsant effect and neurotoxicity of the compounds were calculated with maximal electroshock (MES) test and rotarod tests with intraperitoneally injected mice. Among the synthesized compounds, compound 3-heptyloxy-4-(4-(hexyloxy) phenyl)-4*H*-1,2,4-triazole (5f) was the most active one and also had the lowest toxicity. In the anti-MES potency test, it showed median effective dose (ED_50_) of 37.3 mg/Kg, median toxicity dose (TD_50_) of 422.5 mg/Kg, and the protective index (PI) of 11.3 which is much greater than the reference drug carbamazepine with PI value of 6.4. As well as demonstrating the anti-MES efficacy of compound 5f, its potency against seizures induced by pentylenetetrazole, 3-mercaptopropionic acid, and bicuculline were also established, with the results suggesting that GABA-mediated mechanisms might be involved in its anticonvulsant activity, such as enhancing of GABAergic neurotransmission or activity, activate GAD or inhibit GABA-T, and GABA_A_-mediated mechanisms.

## Introduction

Epilepsy, a ubiquitous disease characterized by recurrent seizures. For epilepsy treatment, nearly 95% of clinically available drugs were approved before 1985 and they are only effective in reducing the severity and number of seizures in less than 70% of patients. These drugs, however, also cause notable adverse side effects such as drowsiness, ataxia, gastrointestinal disturbance, hepatotoxicity and megaloblastic anemia (1-3). Research to find more effective and safer antiepileptic drugs are, therefore, imperative and challenging in medicinal chemistry.

**Figure 1:**
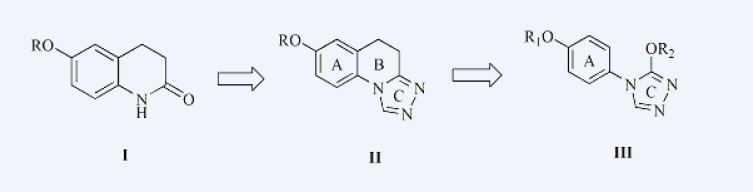
Structure of compounds I, II and III

In our previous work, a series of derivatives of 6-alkoxy-3,4-dihydroquinolin-2(1*H*) -one (Figure 1, compound I) were first found to have anticonvulsant activities(4), with an ED_50_ value of 29.6 mg/Kg in the MES and a TD_50_ value of greater than 300 mg/Kg. Introduction of triazole ring to the first and second position of the compound I caused a remarkable increase in the anticonvulsant activity, series of 6-alkoxy-2(1*H*) quinolones, 1-substituted-7-benzyloxy-4,5-dihydro-[1,2,4]triazolo[4,3-a]quinolines and 7-alkoxy-4,5-dihydro-[1,2,4]triazolo[4,3-a]quinolines (Figure 1, compound II) were synthesized and tested for anticonvulsant activity. (5, 6) 

Analyzing the relationship of anticonvulsant activity and the structure of compound II, it was found that triazole ring C may be the main structure combined with receptor, because the 1,2,4-triazole nucleus has been incorporated into a wide variety of therapeutically important agents, such as antimicrobial (7, 8), anticonvulsant (9, 10) and enzyme inhibition activities (11, 12). And in previous study in our laboratory, we have reported the synthesis and anticonvulsant activity evaluation of some compounds containing triazole, the majority of them exhibited potent anticonvulsant activity (13-19). Aromatic ring A and 7-alkoxyl enhanced the hydrophobic ability of target compounds, thus make them more permeable to the blood-brain barrier and enhance anticonvulsant activity. So we thought that the presence of A and C ring was essential structure for the anticonvulsant activity. To study the effect of ring B opened to the anticonvulsant of compound II, we designed and synthesized a series of derivatives with ring B opened, left the ring A with the best substituents, hexyloxy and heptyloxy (20, 21) and introduction of different alkoxy to ring C. those are the target compounds of this paper, 3-alkoxy-4-(4-(hexyloxy/heptyloxy)phenyl)-4*H*-1,2,4 -triazole (compound III). The hypothesis was that a rotatable triazole ring may have higher affinity for the receptor and enhance their anticonvulsant activity.

## Experimental

*Chemistry*

*General methods*

Melting points were determined in open capillary tubes and are uncorrected. IR spectra were recorded (in KBr) on a FT-IR1730 (Perkin-Elmer, USA). ^1^H-NMR spectra were measured on a AV-300 (Bruker, Switzerland), and all chemical shifts were given in ppm relative to tetramethy-silane. Mass spectra were measured on an HP1100LC (Agilent Technologies, USA). Elemental analyses were performed on a 204Q CHN (Perkin Elmer, USA). Micro-analyses of C, N, and H were performed using a Heraeus CHN Rapid Analyzer. Critical chemicals were purchased from Aldrich Chemical Corporation. All other chemicals were of analytical grade.

*Synthesis of N-(4-(hexyloxy/heptyloxy) phenyl) acetamide (* *2a* */2b)*

A solution of *N*-(4-hydroxyphenyl)acetamide (10 mmol) and K_2_CO_3_ (15 mmol) reacted with appropriate bromohexane/bromoheptane to obtain compound 2a/2b in absolute ethanol with stirring at 80 ℃for 15 hours. Then the solution was evaporated to dryness under reduced pressure, and 20 mL water was poured into the flask, and the mixture was stirred for 0.5 h to eliminate excess K_2_CO_3. _The crude product was collected through filtration and dried in vacuo.

*Synthesis of 4-(hexyloxy/heptyloxy)benzenamine (* *3a* */3b)*

A mixture of N-(4-(hexyloxy/heptyloxy) phenyl) acetamide (10 mmol) and about 30 mL of hydrochloric acid (15 % water solutions) was refluxed for 8 h. Adjusting the solution’s pH value to 8-9 using saturated NaOH resulted in precipitation of solid. The crude product was collected through filtration and dried in vacuo

*Synthesis of 4-(4-(hexyloxy/heptyloxy)phenyl)-2H-1,2,4-triazol-3(4H)-one (* *4a* */4b)*

4-(hexyloxy/heptyloxy) benzenamine (10 mmol), triethyl orthoformate (15 mmol) and methyl hydrazinocarboxylate (15 mmol) were placed into a round-bottomed flask containing 30 mL ethanol. The mixture was refluxed for 24 hours. Then 12 mmol sodium methoxide was put into the flask and continues to be refluxed for 10 hours. After being cooled to the room temperature and concentrated under reduced pressure, the product 4a/4b was collected through filtration.

*General procedure for the synthesis of 3-alkoxy-4-(4-(hexyloxy/heptyloxy)* *phenyl) -4H -1,2,4-triazole (5a-5t)*

4- (4- (hexyloxy/heptyloxy) phenyl) -2H-1,2,4-triazol- 3(4*H*) -one was placed into a 100 mL round-bottomed flask containing 30 mL 15 % NaOH water solutions, stirring at room temperature for half an hour. Then 25 mL ethanol and appropriate alkyl halide (13 mmol) were added to the flask with refluxing and stirring for 7-15 hours (TLC moni-toring). After removing most of the solvent under reduced pressure, the crude product was collected through filtration and recrystallized in acetone to obtain the pure product.

The yield, melting point, analytical data and spectral data of each compound are given below.

*3-* *E* *thoxy-4-(4-(hexyloxy)phenyl)-4H-1,2,4-triazole* *5a*

Yield: 10.3%, mp: 121-123 ^o^C. ^1^H-NMR (CDCl_3_, 300 MHz): *δ* 0.93 (t, 3H, *J* = 6.45 Hz, -CH_3_), 0.99 (t, 3H, *J *= 7.51 Hz, -CH_3_), 1.27-1.47 (m, 4H, -CH_2_-), 1.78-1.86 (m, 4H, -CH_2_-), 3.83 (t, 2H, *J* = 6.90 Hz, -OCH_2_-), 3.97 (q, 2H, *J* = 6.50 Hz -OCH_2_-), 6.98 (dd, 2H, *J* = 8.40 Hz, Ar-H), 7.43 (dd, 2H, *J* = 8.40 Hz, Ar-H), 7.62 (s, 1H, -N=CH-), IR (KBr) cm^-1^: 2968 (-N=CH-), 1683 (-R-O-C-); MS-EI *m/z* 290 (M+1). 

*3-* *P* *ropoxy-4-(4-(hexyloxy)phenyl)-4H-1,2,4-triazole* *5b*

Yield: 11.7%, mp: 115-117 ^o^C. ^1^H-NMR (CDCl_3_, 300 MHz): *δ* 0.92 (t, 3H, *J* = 6.35 Hz, -CH_3_), 1.01 (t, 3H, *J* = 8.85 Hz, -CH_3_), 1.35-1.47 (m, 6H, -CH_2_-), 1.78-1.90 (m, 4H, -CH_2_-), 3.82 (t, 2H, *J* = 6.75 Hz, -OCH_2_-), 3.96 (t, 2H, *J* = 7.05 Hz, -OCH_2_-), 6.97 (dd, 2H, *J* = 7.50 Hz, Ar-H), 7.42 (dd, 2H, *J* = 7.50 Hz, Ar-H), 7.62 (s, 1H, -N=CH-), IR (KBr) cm^-1^: 2969 (-N=CH-), 1685 (-R-O-C-); MS-EI *m/z* 304 (M+1).

*3-* *B* *utoxy-4-(4-(hexyloxy)phenyl)-4H-1,2,4-triazole* *5c*

Yield: 17.6%, mp: 111-113 ^o^C. ^1^H-NMR (CDCl_3_, 300 MHz): *δ* 0.89 (t, 3H, *J* = 6.45 Hz, -CH_3_), 0.96 (t, 3H, *J* = 7.35 Hz, -CH_3_), 1.31-1.45 (m, 8H, -CH_2_-), 1.70-1.93 (m, 4H, -CH_2_-), 3.84 (t, 2H, *J* = 7.05 Hz, -OCH_2_-), 3.96 (t, 2H, *J* = 6.45 Hz, -OCH_2_-), 6.95 (dd, 2H, *J* = 8.70 Hz, Ar-H), 7.37 (dd, 2H, *J* = 8.70 Hz, Ar-H), 7.62 (s, 1H, -N=CH-), IR (KBr) cm^-1^: 2967 (-N=CH-), 1684 (-R-O-C-); MS-EI *m/z* 318 (M+1).

*3-* *P* *entyloxy-4-(4-(hexyloxy)phenyl)-4H-1,2,4-triazole* *5d*

Yield: 18.3%, mp: 129-131 ^o^C. ^1^H-NMR (CDCl_3_, 300 MHz): *δ* 0.90 (t, 3H, *J* = 6.15 Hz, –CH_3_), 1.01 (t, 3H, *J* = 6.80 Hz, –CH_3_), 1.27-1.35 (m, 8H, -CH_2_-), 1.38-1.85 (m, 6H, -CH_2_-), 3.86 (t, 2H, *J* = 7.20 Hz, -OCH_2_-), 3.99 (t, 2H, *J* = 6.30 Hz, -OCH_2_-), 6.98 (dd, 2H, *J* = 9.00 Hz, Ar-H), 7.43 (dd, 2H, *J* = 9.00 Hz, Ar-H), 7.62 (s, 1H, -N=CH-), IR (KBr) cm^-1^: 2969 (-N=CH-), 1685 (-R-O-C-); MS-EI *m/z* 332 (M+1).

*3-* *H* *exyloxy-4-(4-(hexyloxy)phenyl)-4H-1,2,4-triazole* *5e*

Yield: 20.6%, mp: 136-138 ^o^C. ^1^H-NMR (CDCl_3_, 300 MHz): *δ* 0.84 (t, 3H, *J* = 6.04 Hz, -CH_3_), 0.92 (t, 3H, *J* = 7.15 Hz, -CH_3_), 0.97-1.42 (m, 10H, -CH_2_-), 1.44-1.86 (m, 6H, -CH_2_-), 3.86 (t, 2H, *J* = 7.20 Hz, -OCH_2_-), 3.99 (t, 2H, *J* = 6.60 Hz, -OCH_2_-), 6.98 (dd, 2H, *J* = 8.70 Hz, Ar-H), 7.42 (dd, 2H, *J* = 8.70 Hz, Ar-H), 7.62 (s, 1H, -N=CH-), IR (KBr) cm^-1^: 2969 (-N=CH-), 1687 (-R-O-C-); MS-EI *m/z* 346 (M+1). 

*3-* *H* *eptyloxy-4-(4-(hexyloxy)phenyl)-4H-1,2,4-triazole* *5f*

Yield: 46.8%, mp: 137-139 ^o^C. ^1^H-NMR (CDCl_3_, 300 MHz): *δ* 0.89 (t, 3H, *J* = 5.85 Hz, -CH_3_), 0.93 (t, 3H, *J* = 5.55 Hz, -CH_3_), 1.21-1.48 (m, 14H, -CH_2_-), 1.76-1.85 (m, 4H, -CH_2_-), 3.85 (t, 2H, *J* = 7.20 Hz, -OCH_2_-), 3.97 (t, 2H, *J* = 6.60 Hz, -OCH_2_-), 6.98 (dd, 2H, *J* = 9.00 Hz, Ar-H), 7.42 (dd, 2H, *J* = 9.00 Hz, Ar-H), 7.62 (s, 1H, -N=CH-), IR (KBr) cm^-1^: 2965 (-N=CH-), 1686 (-R-O-C-); MS-EI *m/z* 360 (M+1). 

*3-* *O* *ctyloxy-4-(4-(hexyloxy)phenyl)-4H-1,2,4-triazole* *5g*

Yield: 46.8%, mp: 139-141 ^o^C. ^1^H-NMR (CDCl_3_, 300 MHz): *δ* 0.90 (t, 3H, *J* = 4.50 Hz, -CH_3_), 0.93 (t, 3H, *J* = 9.00 Hz, -CH_3_), 1.27-1.47 (m, 16H, -CH_2_-), 1.49-1.83 (m, 4H, -CH_2_-), 3.85 (t, 2H, *J* = 7.20 Hz, -OCH_2_-), 3.97 (t, 2H, *J* = 6.30 Hz, -OCH_2_-), 6.98 (dd, 2H, *J* = 8.70 Hz, Ar-H), 7.42 (dd, 2H, *J* = 8.70 Hz, Ar-H), 7.62 (s, 1H, -N=CH-), IR (KBr) cm^-1^: 2964 (-N=CH-), 1688 (-R-O-C-); MS-EI *m/z* 374 (M+1). 

*3-* *D* *ecyloxy-4-(4-(hexyloxy)phenyl)-4H-1,2,4-triazole* *5h*

Yield: 45.4%, mp: 123-125 ^o^C. ^1^H-NMR (CDCl_3_, 300 MHz): *δ* 0.89 (t, 3H, *J* = 5.35 Hz, -CH_3_), 0.93 (t, 3H, *J* = 9.00 Hz, -CH_3_), 1.27-1.48 (m, 20H, -CH_2_-), 1.50-1.85 (m, 4H, -CH_2_-), 3.85 (t, 2H, *J* = 6.30 Hz, -OCH_2_-), 3.99 (t, 2H, *J* = 7.20 Hz, -OCH_2_-), 6.98 (dd, 2H, *J* = 8.70 Hz, Ar-H), 7.42 (dd, 2H, *J* = 8.70 Hz, Ar-H), 7.62 (s, 1H, -N=CH-), IR (KBr) cm^-1^: 2969 (-N=CH-), 1687 (-R-O-C-); MS-EI *m/z* 402 (M+1).

*3-* *D* *odecyloxy-4-(4-(hexyloxy)phenyl)-4H-1,2,4-triazole* *5i*

Yield: 48.8%, mp: 115-117 ^o^C. ^1^H-NMR (CDCl_3_, 300 MHz): *δ* 0.89 (t, 3H, *J* = 5.70 Hz, -CH_3_), 0.92 (t, 3H, *J* = 6.80 Hz, -CH_3_), 1.19-1.37 (m, 24H, -CH_2_-), 1.43-1.52 (m, 4H, -CH_2_-), 3.85 (t, 2H, *J* = 7.20 Hz, -OCH_2_-), 3.98 (t, 2H, *J* = 6.60 Hz, -OCH_2_-), 6.98 (dd, 2H, *J* = 9.00 Hz, Ar-H), 7.42 (dd, 2H, *J* = 9.00 Hz, Ar-H), 7.62 (s, 1H, -N=CH-), IR (KBr) cm^-1^: 2968 (-N=CH-), 1685 (-R-O-C-); MS-EI *m/z* 430 (M+1). 

*3-* *B* *enzyloxy-4-(4-(hexyloxy)phenyl)-4H-1,2,4-triazole* *5j*

Yield: 48.8%, mp: 165-167 ^o^C. ^1^H-NMR (CDCl_3_, 300 MHz): *δ* 0.93 (t, 3H, *J* = 7.15 Hz, -CH_3_), 0.93 (t, 3H, *J* = 8.05 Hz, -CH_3_), 1.35-1.50 (m, 6H, -CH_2_-), 1.77-1.87 (m, 2H, -CH_2_-), 3.98 (t, 2H, *J* = 7.80 Hz, -OCH_2_-), 5.04 (s, 2H, -OCH_2_-), 6.96-7.04 (m, 2H, Ar-H), 7.32-7.62 (m, 7H, Ar-H), 7.64 (s, 1H, -N=CH-), IR (KBr) cm^-1^: 2964 (-N=CH-), 1689 (-R-O-C-); MS-EI *m/z* 352 (M+1). 

*3-* *E* *thoxy-4-(4-(heptyloxy)phenyl)-4H-1,2,4-triazole* *5k*

Yield: 10.5%, mp: 121-123 ^o^C. ^1^H-NMR (CDCl_3_, 300 MHz): *δ* 0.87 (t, 3H, *J* = 6.15 Hz, -CH_3_), 0.93 (t, 3H, *J* = 7.82 Hz, -CH_3_), 1.31-1.74 (m, 6H, -CH_2_-), 1.76-1.81 (m, 4H, -CH_2_-), 3.87 (t, 2H, *J* = 7.35 Hz, -OCH_2_-), 3.95 (q, 2H, *J* = 6.55 Hz -OCH_2_-), 6.96 (dd, 2H, *J* = 4.35 Hz, Ar-H), 7.40 (dd, 2H, *J* = 4.35 Hz, Ar-H), 7.60 (s, 1H, -N=CH-), IR (KBr) cm^-1^: 2965 (-N=CH-), 1686 (-R-O-C-); MS-EI *m/z* 304 (M+1). 

*3-* *P* *ropoxy-4-(4-(heptyloxy)phenyl)-4H-1,2,4-triazole* ***5l***

Yield: 16.4%, mp: 127-129 ^o^C. ^1^H-NMR (CDCl_3_, 300 MHz): *δ* 0.89 (t, 3H, *J* = 6.45 Hz, -CH_3_), 0.93 (t, 3H, *J* = 7.35 Hz, -CH_3_), 1.21-1.48 (m, 14H, -CH_2_-), 1.76-1.85 (m, 4H, -CH_2_-), 3.85 (t, 2H, *J* = 6.90 Hz, -OCH_2_-), 3.97 (t, 2H, *J* = 6.45 Hz, -OCH_2_-), 6.98 (dd, 2H, *J* = 8.40 Hz, Ar-H), 7.42 (dd, 2H, *J* = 8.40 Hz, Ar-H), 7.62 (s, 1H, -N=CH-), IR (KBr) cm^-1^: 2967 (-N=CH-), 1687 (-R-O-C-); MS-EI *m/z* 318 (M+1). 

*3-* *B* *utoxy-4-(4-(heptyloxy)phenyl)-4H-1,2,4-triazole* *5m*

Yield: 15.3%, mp: 133-135 ^o^C. ^1^H-NMR (CDCl_3_, 300 MHz): *δ* 0.89 (t, 3H, *J* = 6.45 Hz, -CH_3_), 0.96 (t, 3H, *J* = 7.05 Hz, -CH_3_), 1.31-1.43 (m, 10H, -CH_2_-), 1.69-1.80 (m, 4H, -CH_2_-), 3.84 (t, 2H, *J* = 6.45 Hz, -OCH_2_-), 3.96 (t, 2H, *J* = 6.45 Hz, -OCH_2_-),6.95 (dd, 2H, *J* = 8.70 Hz, Ar-H), 7.39 (dd, 2H, *J* = 8.70 Hz, Ar-H), 7.62 (s, 1H, -N=CH-), IR (KBr) cm^-1^: 2969 (-N=CH-), 1684 (-R-O-C-); MS-EI *m/z* 332 (M+1). 

*3-* *P* *entyloxy-4-(4-(heptyloxy)phenyl)-4H-1,2,4-triazole* *5n*

Yield: 16.4%, mp: 145-147 ^o^C. ^1^H-NMR (CDCl_3_, 300 MHz): *δ* 0.90 (t, 3H, *J* = 4.65 Hz, -CH_3_), 0.93 (t, 3H, *J* = 4.50 Hz, -CH_3_), 1.07-1.47 (m, 10H, -CH_2_-), 1.65-1.85 (m, 6H, -CH_2_-), 3.85 (t, 2H, *J* = 6.90 Hz, -OCH_2_-), 3.99 (t, 2H, *J* = 6.15 Hz, -OCH_2_-), 6.98 (dd, 2H, *J* = 8.10 Hz, Ar-H), 7.42 (dd, 2H, *J* = 8.10 Hz, Ar-H), 7.62 (s, 1H, -N=CH-), IR (KBr) cm^-1^: 2968 (-N=CH-), 1684 (-R-O-C-); MS-EI *m/z* 346 (M+1). 

*3-* *H* *exyloxy-4-(4-(heptyloxy)phenyl)-4H-1,2,4-triazole* *5o*

Yield: 26.7%, mp: 149-151 ^o^C. ^1^H-NMR (CDCl_3_, 300 MHz): *δ* 0.79 (t, 3H, *J* = 6.85 Hz, -CH_3_), 0.98 (t, 3H, *J* = 6.05 Hz, -CH_3_), 1.32-1.73 (m, 14H, -CH_2_-), 1.75-1.80 (m, 4H, -CH_2_-), 3.83 (t, 2H, *J* = 7.20 Hz, -OCH_2_-), 3.96 (t, 2H, *J* = 6.45 Hz, -OCH_2_-), 6.94 (dd, 2H, *J* = 9.00 Hz, Ar-H), 7.40 (dd, 2H, *J* = 9.00 Hz, Ar-H), 7.60 (s, 1H, -N=CH-), IR (KBr) cm^-1^: 2970 (-N=CH-), 1689 (-R-O-C-); MS-EI *m/z* 360 (M+1). 

*3-* *H* *eptyloxy-4-(4-(heptyloxy)phenyl)-4H-1,2,4-triazole* *5p*

Yield: 36.5%, mp: 156-158 ^o^C. ^1^H-NMR (CDCl_3_, 300 MHz): *δ* 0.85 (t, 3H, *J* = 6.75 Hz, -CH_3_), 0.90 (t, 3H, *J* = 6.45 Hz, -CH_3_), 1.03-1.40 (m, 14H, -CH_2_-), 1.42-1.81 (m, 6H, -CH_2_-), 3.84 (t, 2H, *J* = 7.20 Hz, -OCH_2_-), 3.97 (t, 2H, *J* = 6.60 Hz, -OCH_2_-), 6.96 (dd, 2H, *J* = 9.00 Hz, Ar-H), 7.41 (dd, 2H, *J* = 9.00 Hz, Ar-H), 7.60 (s, 1H, -N=CH-), IR (KBr) cm^-1^: 2971 (-N=CH-), 1682 (-R-O-C-); MS-EI *m/z* 374 (M+1). 

*3-* *O* *ctyloxy-4-(4-(heptyloxy)phenyl)- 4H-1,2,4-triazole* *5q*

Yield: 31.2%, mp: 167-169 ^o^C. ^1^H-NMR (CDCl_3_, 300 MHz): *δ* 0.85 (t, 3H, *J* = 6.35 Hz, -CH_3_), 0.92 (t, 3H, *J* = 6.60 Hz, -CH_3_), 1.27-1.69 (m, 16H, -CH_2_-), 1.71-1.84 (m, 6H, -CH_2_-), 3.84 (t, 2H, *J* = 6.45 Hz, -OCH_2_-), 3.97 (t, 2H, *J* = 6.45 Hz, -OCH_2_-), 6.96 (dd, 2H, *J* = 9.00 Hz, Ar-H), 7.41 (dd, 2H, *J* = 9.00 Hz, Ar-H), 7.60 (s, 1H, -N=CH-), IR (KBr) cm^-1^: 2972 (-N=CH-), 1670 (-R-O-C-); MS-EI *m/z* 388 (M+1). 

*3-* *D* *ecyloxy-4-(4-(heptyloxy)phenyl)-4H-1,2,4-triazole* *5r*

Yield: 40.8%, mp: 147-149 ^o^C. ^1^H-NMR (CDCl_3_, 300 MHz): *δ* 0.81 (t, 3H, *J* = 5.97 Hz, -CH_3_), 0.97 (t, 3H, *J* = 15.0 Hz, -CH_3_), 1.15-1.53 (m, 18H, -CH_2_-), 1.72-1.92 (m, 8H, -CH_2_-), 3.83 (t, 2H, *J* = 6.75 Hz, -OCH_2_-), 3.97 (t, 2H, *J* = 5.55 Hz, -OCH_2_-), 6.96 (dd, 2H, *J* = 8.70 Hz, Ar-H), 7.40 (dd, 2H, *J* = 8.70 Hz, Ar-H), 7.60 (s, 1H, -N=CH-), IR (KBr) cm^-1^: 2971 (-N=CH-), 1671 (-R-O-C-); MS-EI *m/z* 416 (M+1).

*3-* *D* *odecyloxy-4-(4-(heptyloxy)phenyl)-4H-1,2,4-triazole* *5s*

Yield: 39.2%, mp: 152-154 ^o^C. ^1^H-NMR (CDCl_3_, 300 MHz): *δ* 0.85 (t, 3H, *J* = 6.90 Hz, -CH_3_), 0.91 (t, 3H, *J* = 6.90 Hz, -CH_3_), 1.10-1.48 (m, 26H, -CH_2_-), 1.68-1.83 (m, 4H, -CH_2_-), 3.83 (t, 2H, *J* = 7.20 Hz, -OCH_2_-), 3.97 (t, 2H, *J* = 6.60 Hz, -OCH_2_-), 6.96 (dd, 2H, *J* = 9.00 Hz, Ar-H), 7.40 (dd, 2H, *J* = 9.00 Hz, Ar-H), 7.60 (s, 1H, -N=CH-), IR (KBr) cm^-1^: 2970 (-N=CH-), 1673 (-R-O-C-); MS-EI *m/z* 444 (M+1). 

*3-* *B* *enzyloxy-4-(4-(heptyloxy)phenyl)-4H-1,2,4-triazole* *5t*

Yield: 46.4%, mp: 168-169 ^o^C. ^1^H-NMR (CDCl_3_, 300 MHz): *δ* 0.90 (t, 3H, *J* = 6.15 Hz, -CH_3_), 0.91 (t, 3H, *J* = 6.95 Hz, -CH_3_), 1.05-1.46 (m, 6H, -CH_2_-), 1.44-1.83 (m, 4H, -CH_2_-), 3.97 (t, 2H, *J* = 6.45 Hz, -OCH_2_-), 5.02 (s, 2H, -OCH_2_-), 6.95-7.07 (m, 2H, Ar-H), 7.31-7.42 (m, 7H, Ar-H), 7.60 (s, 1H, -N=CH-), IR (KBr) cm^-1^: 2974 (-N=CH-), 1686 (-R-O-C-); MS-EI *m/z* 366 (M+1). 

*Pharmacology*

The MES test, Chemicals induce epileptic test and rotarod test were carried out according to the procedures described in Anticonvulsant Screening Program with some modification (22, 23). In the MES test and rotarod test, the anticonvulsant effects and the neurovirulence of the compounds were assessed at 0.5 h intervals following administration in mice. And in preliminary neurotoxicity screening, compounds only were administered by intraperitoneal (*i.p*.) injection at dosages of 100mg/kg to avoid wasting animals. Electro-convulsions were produced by an electric stimulation generator (JTC-1, ChengDu, China). All compounds, which were dissolved in dimethylsulfoxide (DMSO), were evaluated for anticonvulsant activities with KunMing mice in the 18-22 g weight range purchased from the Laboratory of Animal Research, College of Pharmacy, Yanbian University. 

*The* *maximal electroshock (MES)* *test*

Seizures were elicited with a 60 Hz alternating current of 50 mA intensity in mice. The current was applied *via* ear-clip electrodes for 0.2s. Protection against the spread of MES-induced seizures was defined as the absence of tonic extension of the hind leg. At 30 min after the administration of the compounds, the activities were evaluated in MES test. In preliminary screening, each compound was administered at the dose levels of 200, 100，and 30 mg/Kg for evaluating the preliminary anticonvulsant activity. For determination of the median effective dose (ED_50_) and the median toxic dose (TD_50_), the quantitative evaluation was prepared. Groups of 10 mice were given a range of intraperitoneal doses of the tested compound until at least three points were established in the range of 10–90% seizure protection or neurotoxicity. From these data, the respective ED_50_, TD_50_ values, and 95% confidence intervals were calculated by probit analysis.

*Rotarod test (* 24 *)*

The neurotoxicity of the compounds was measured in mice by the rotarod test. The mice were trained to stay on an accelerating rotarod of diameter an inch that rotates at 6 rpm. Trained animals were given *i.p*. injection of the test compounds. Neurotoxicity was indicated by the inability of the animal to maintain equilibrium on the rod for at least 1min in each of the trials.

*Chemical model seizure test*

*Pentylenetetrazol* *-induced seizure test (* 25 *)*

At 30 min after the administration of the test compound, 100 mg/Kg of PTZ dissolved in saline was administered sc. The animals placed in individual cages and observed for 30 min. The number of clonic and tonic seizures as well as the number of deaths was noted.

*Mercaptopropionic acid* *-induced seizures (* 26 *)*

At 30 min after the administration of the test compound, 40 mg/Kg of 3-MP dissolved in saline solution was injected sc. The animals placed in individual cages and observed for 30 min. The number of clonic and tonic seizures as well as the number of deaths was noted.

*Bicuculline-induced seizures test (* 27 *)*

At 30 min after the administration of the test compound, 5.4 mg/Kg of bicuculline (within 15-45 min after preparation due to instability) was injected sc. The animals placed in individual cages and observed for 30 min. The number of clonic and tonic seizures as well as the number of deaths was noted.

*C* *alculation formula*

ED_50 _= lg^-1 ^[Xm - i(∑P-0.5)]

S_X50 _=i [(∑P -∑P)^2 ^/n-1]^1/2^

The 95% confidence limits= lg^-1^(lgED_50 _±1.96S_X50_)

The meaning of the symbol in the formula:

Xm: Maximum dose of logarithm.

i: Dose the proportion between the value of common logarithm.

P: The positive rate of each group.

n: The number of animal in each group.

S_X50_: The standard error of lgED_50_. 

TD_50_ has the same calculation method as above.

## Results and Discussion

*Chemistry*

The structures of those new compounds were characterized using IR,^ 1^H-NMR, MS and elemental analysis techniques. Their anticonvulsant activity was evaluated by the maximal electroshock test (MES) and their neurotoxicity was evaluated by the rotarod test. To elucidate the possible mechanism of action, the most active compound was tested in pentylenetetrazole (PTZ), 3-mercaptopropionic acid (3-MP), and bicuculline (BIC) induced seizure tests.

**Figure 2:**
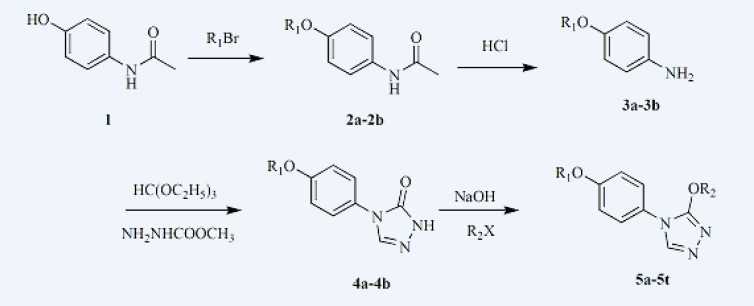
Synthetic route of target compounds (5a-5t).

Based on the previous studies in our laboratory, we designed and synthesized a series of 3-alkoxy-4-(4-(hexyloxy/heptyloxy)phenyl)-4*H*-1,2,4-triazole (5a-5t). Target compounds were prepared along the reaction sequence in Figure 2. Starting material N-(4-hydroxyphenyl)acetamide reacted with appropriate bromohexane or bromoheptane to obtain compound 2a or 2b in ethanol with stirring and refluxing (28). 2a or 2b then reacted with hydrochloric acid (15 % water solutions) to obtain the compound 3a or 3b. 3a or 3b reacted further with triethyl orthoformate and methyl hydrazinocarboxylate in ethanol and then put appropriate sodium methoxide into the mixture to obtain compound 4a or 4b (29, 30). At last, derivatives 5a-5t were obtained through an alkylation reaction of compound 4a or 4b with appropriate alkyl halide, respectively (28).

*Pharmacological evaluations*

During the preliminary screening process, compounds were administered by intraperitoneal (*i.p*.) injection at dosages of 200, 100, and 30 mg/Kg. Their anticonvulsant effects and neurovirulence were assessed at 0.5 h intervals following administration in mice.

The results of preliminary (phase I) screening of 5a-5t are summarized in Table 1. Some synthesized compounds exhibited anticonvulsant activities, among which two compounds, 5f and 5p, showed partial protection (1/3 or 2/3) at a dose of 30 mg/Kg, while others displayed a little neurovirulence at a dosage of 100 mg/Kg, but not so strong.

**Table 1:** Preliminary screening of anticonvulsant activity in mice (i.p.).

**Compounds**	**R**_1_	**R**_2_	**MES(mg/Kg)** a ^,^ b		
			**200**	**100**	**30**
**5a**	*n*-C_6_H_13_	*n*-C_2_H_5_	0/3	0/3	0/3
**5b**	*n*-C_6_H_13_	*n*-C_3_H_7_	0/3	0/3	0/3
**5c**	*n*-C_6_H_13_	*n*-C_4_H_9_	0/3	0/3	0/3
**5d**	*n*-C_6_H_13_	*n*-C_5_H_11_	0/3	0/3	0/3
**5e**	*n*-C_6_H_13_	*n*-C_6_H_13_	2/3	1/3	0/3
**5f**	*n*-C_6_H_13_	*n*-C_7_H_15_	-	3/3	2/3
**5g**	*n*-C_6_H_13_	*n*-C_8_H_17_	3/3	2/3	0/3
**5h**	*n*-C_6_H_13_	*n*-C_10_H_21_	1/3	0/3	0/3
**5i**	*n*-C_6_H_13_	*n*-C_12_H_25_	0/3	0/3	0/3
**5j**	*n*-C_6_H_13_	*n*-CH_2_C_6_H_5_	2/3	1/3	0/3
**5k**	*n*-C_7_H_15_	*n*-C_2_H_5_	0/3	0/3	0/3
**5l**	*n*-C_7_H_15_	*n*-C_3_H_7_	0/3	0/3	0/3
**5m**	*n*-C_7_H_15_	*n*-C_4_H_9_	1/3	0/3	0/3
**5n**	*n*-C_7_H_15_	*n*-C_5_H_11_	1/3	0/3	0/3
**5o**	*n*-C_7_H_15_	*n*-C_6_H_13_	2/3	1/3	0/3
**5p**	*n*-C_7_H_15_	*n*-C_7_H_15_	3/3	2/3	1/3
**5q**	*n*-C_7_H_15_	*n*-C_8_H_17_	1/3	0/3	0/3
**5r**	*n*-C_7_H_15_	*n*-C_10_H_21_	0/3	0/3	0/3
**5s**	*n*-C_7_H_15_	*n*-C_12_H_25_	0/3	0/3	0/3
**5t**	*n*-C_7_H_15_	*n*-CH_2_C_6_H_5_	2/3	1/3	0/3

a Maximal electroshock: doses of 30, 100 and 200 mg/Kg were administrated intraperitoneally in mice. The animals were examined 0.5 h after administration.

b n_1_/n_2_: the animals protected / the animals tested.

*Structure activity relationships*

Following activity analysis of the synthesized compounds, the following structure–activity relationships (SARs) were obtained. For the alkyl chain-substituted derivatives 5a-5i and 5k-5s, the length of the alkyl chain appeared to have a direct impact on the anticonvulsant activity of the derivatives. For example when R_1_ was *n*-hexane chain, as the alkyl chain length increased from 5a to 5f, the anticonvulsant activity gradually increased with compound 5f being the most active compound. From 5f to 5i, although the alkyl chain length increased further, the anticonvulsant activity decreased and ultimately disappeared. When R_1_ was n-heptane chain, as the alkyl chain length increased from 5k to 5p , the anticonvulsant activity gradually increased with compound 5p being the most active compound. From 5p to 5s, although the alkyl chain length increased further, the anticonvulsant activity decreased and ultimately disappeared. This structure-activity relationship might be associated with the lipid-water partition coefficients of the compounds, which affects drug absorption, hydrophobic drug-receptor interactions, metabolism of molecules, and especially the ability to cross the blood-brain barrier (BBB). The compounds 5j and 5t, which substituted with a benzyloxy group, showed less anticonvulsant activity than the best alkyl chain-substituted 5f and 5p**.**

*Quantitative evaluation of the anticonvulsant activity*

Based on the considerable anticonvulsant activity demonstrated during the preliminary screening test, compounds 5f and 5p were subjected to quantitative evaluation trials for quantification of their anticonvulsant activity (indicated by ED_50_) and neurotoxicity (indicated by TD_50_) in mice. Results of the quantitative testing of selected compounds, together with data of the antiepileptic drug carbamazepine (measured in the same condition), are shown in Table 2.

**Table 2:** Quantitative anticonvulsant data in Mice (i.p.).

**Componds**	**R**_1_	**R**_2_	**ED**_50_ **(mg/kg)** **(MES)** a	**TD**_50_ **(mg/kg)** **(MES)** b	**PI** c
**5f**	*n*-C_6_H_13_	*n*-C_7_H_15_	37.3(32.4-43.1)^d^	422.5(366.2-487.4)	11.3
**5p**	*n*-C_7_H_13_	*n*-C_7_H_15_	41.2(40.0-54.6)	352.1(305.2-406.2	8.5
Carbarmazepine	-	-	11.8(9.7-14.1)	76.1(69.1-83.7)	6.5

a : median effective dose affording anticonvulsant protection in 50% of animals, the dose is measured in mg/Kg.

b : median toxic dose eliciting minimal neurological toxicity in 50% of animals, the dose is measured in mg/Kg.

c : protective index (TD_50_/ED_50_).

d : 95% confidence intervals given in parentheses.

In Table 2, 5f and 5p possessed excellent anticonvulsant activity with ED_50_ values of 37.3 and 41.2 mg/Kg, respectively. Compound 5f exhibited stronger activity than compound 5p against seizure induced by MES, in additional, compound 5f possessed lower neurotoxicity with PI value of 11.3. Compared with the reference carbarmazepine, the two compounds 5f and 5p showed possessed lower neurotoxicity.

*Speculation of mechanism*

As a result of the quantitative evaluation, compound 5f showed strong anticonvulsant activity and the best PI value in MES test, so it was then selected for further investigations against seizures induced by pentylenetetrazole, 3-mercaptopropionic acid, and bicuculline to prove its anticonvulsant activity and speculate about the possible mechanism of anticonvulsant action. 

We used ten mice per group in our experiment. Compound 5f and the reference drug carbamazepine were administered to mice at 50 mg/Kg *i.p*., which was higher than its ED_50_ value and far below its TD_50_ value. 

We could see that some compounds that we have synthesized have a notable anticonvulsion activity in MES test, which is related to the effect of compound on ion channels. So we can say that the anticonvulsant activity of some compounds partially through affecting the function of ion channels.

**Table 3:** Effects of compound 5f on chemical-induced seizures in mice (i.p.).

**Chemical substances**	**Compound**	**Doses (mg/Kg)**	**Test time (h)**	**Clonic seizures (%)**	**Tonic seizures** ** (%) **	**Lethality** **(%)**
Pentylenetetrazol	DMSO	-	0.5	100	100	100
	Carbamazepine	50	0.5	100	0	10
	5f	50	0.5	100	40	50
						
3-Mercaptopropionic	DMSO	-	0.5	100	100	100
acid	Carbamazepine	50	0.5	100	20	10
	5f	50	0.5	100	20	0
						
Bicuculline	DMSO	-	0.5	100	100	100
	Carbamazepine	50	0.5	100	0	60
	5f	50	0.5	100	0	20

PTZ has been reported to produce seizures by inhibiting gamma-aminobutyic acid (GABA) neurotransmission (31). GABA is the main inhibitory neurotransmitter substance in the brain, and is widely implicated in epilepsy. Inhibition of GABAergic neurotransmission or activity has been shown to promote and facilitate seizures (32), while enhancement of GABAergic neurotransmission is known to inhibit or attenuate seizures. In the sc-PTZ model, compound 5f inhibited the clonic seizures, tonic seizures and lethality at the rates of 0%, 60%, and 50% induced by sc-PTZ, respectively (Table 3). The findings of the present study tend to suggest that compound 5f might have inhibited or attenuated PTZ-induced seizure in mice by enhancing GABAergic neurotransmission.

3-MP acid was seen as the competitive inhibitor of GABA synthesis enzyme glutamate decarboxylase (GAD), inhibited the synthesis of GABA to decrease the GABA level in the brain (33). In the 3-MP induced seizure model, carbamazepine inhibited the clonic seizures, tonic seizures and death at rates of 0%, 80%, and 90%, respectively. By comparison, compounds 5f showed an anticonvulsant effect similar to that of carbamazepine in inhibiting the clonic and tonic seizures, showed a little better in inhibiting lethality (Table 3). The moderate antagonism of 3-MP-induced seizure suggests that compound 5f might activate GAD or inhibit (GABA)-a-oxoglutarate aminotransferase (GABA-T) in the brain.

In the BIC induced seizure model, both carbamazepine and 5f inhibited tonic seizures and death, but did not inhibit clonic seizures. Carbamazepine showed inhibition of clonic and tonic seizures and death at rates of 0%, 100%, and 40%, respectively. Compound 5f inhibited the clonic seizures, tonic seizures and lethality at the rates of 0%, 100%, and 80% induced by BIC, respectively, better than carbamazepine in inhibiting lethality (Table 3). BIC is a competitive antagonist of the GABA_A _receptor (34). As compound 5f inhibited the tonic seizures induced by BIC, it likely exerts anticonvulsant activity, at least partially, through GABA_A_-mediated mechanisms. 
